# Supplementary material for: SynOmics: integrating multi-omics data through feature interaction networks
Source: Brief Bioinform. 2025 Nov 13;26(6):bbaf595. doi: 10.1093/bib/bbaf595 (PMC12613832; doi:10.1093/bib/bbaf595)
Supplement: Supplement_bbaf595 [file supplement_bbaf595.pdf]

S1 Survival Analysis

Figure S1 illustrates the survival prediction performance derived from the original datasets for breast (BRCA), lung (LUAD), and ovarian (OV) cancers.

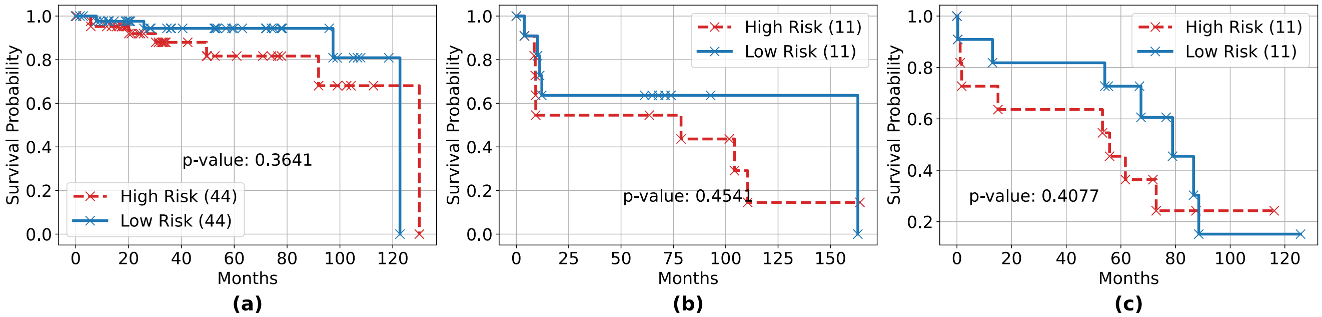

Figure S1: Survival analysis based on original input features for (a) BRCA (ER status), (b) LUAD (survival duration), and (c) OV (survival duration) datasets. The numbers in parentheses indicate the sample sizes for the low- and high-risk groups. The p-value is computed using the log-rank test to assess the difference in overall survival between the two groups.

S2 Reliance on Priors: Perturbation Tests and Mixture Network

We tested SynOmics' reliance on priors by randomly rewiring 10%–50% of edges in the mRNA–miRNA network. As perturbation increased, performance declined gradually (Figure S2): median AUC dropped from 0.669 to 0.603 (–10%), and MCC from 0.325 to 0.273 (–16%). Overall, SynOmics remains robust to incomplete/noisy priors with minor fluctuations, though preserving reasonable prior structure is still beneficial.

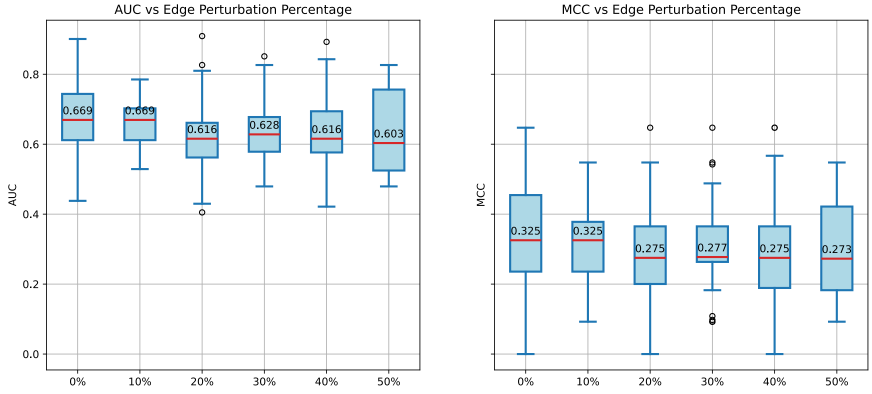

Figure S2: **Robustness of SynOmics under network perturbation.** AUC (left) and MCC (right) are shown as the percentage of randomly perturbed edges in the mRNA–miRNA network increases from 0% to 50%. Boxplots summarize results across 100 data splits; horizontal red bars mark medians (annotated), boxes indicate interquartile ranges.

To further assess the contribution of curated priors, we blended prior and cohort-derived edges for the mRNA–miRNA interaction network using a convex mixture

$$A(\lambda) = \lambda A_{\text{prior}} + (1-\lambda)A_{\text{derived}}, \qquad \lambda \in [0,1],$$

where A_{derived} is built via cosine similarity. As observed in Figure S3, performance remains broadly stable across λ, with small but consistent gains as reliance on the curated prior increases: the median AUC rises from 0.645 at $\lambda = 0$ (data-only) to 0.665 at $\lambda = 1$ (prior-only), a $\sim$3.1% improvement. These results support the feasibility of hybrid graphs and suggest that, when high-quality priors are available, assigning them greater weight is beneficial.

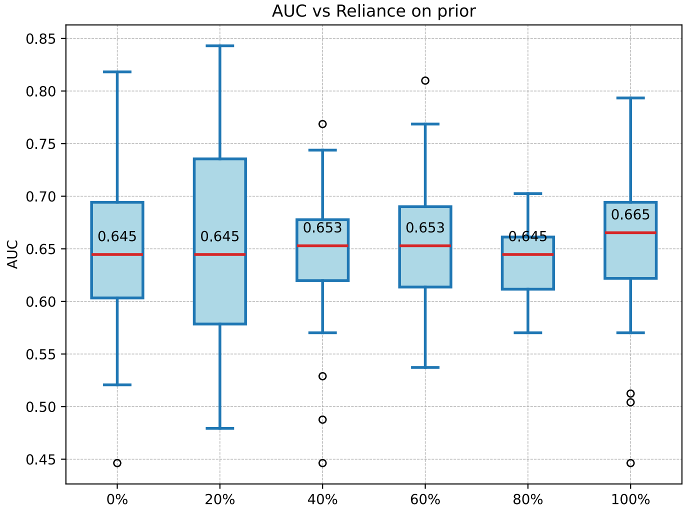

Figure S3: **Effect of prior weight on performance.** Test AUC versus the prior graph weight λ for the mRNA–miRNA interaction network in the hybrid adjacency $A(\lambda) = \lambda A_{\text{prior}} + (1-\lambda)A_{\text{derived}}$. Boxplots summarize results across 100 data splits; horizontal red bars mark medians (annotated), boxes indicate interquartile ranges.

S3 Similarity Choice for Inter-Omics Networks

We compared cosine, Pearson, and a Gaussian-kernel similarity for building the intra- and inter-omics networks (Figure S4). Gaussian performed close to cosine in AUC (0.661 vs. 0.669) and above Pearson (0.649), and improved MCC over Pearson (0.293 vs. 0.277). Cosine remained slightly best overall, likely because it is scale-invariant, emphasizes directional alignment, is robust to mean–variance shifts/outliers across assays, and avoids bandwidth tuning that can over/under-connect nodes for Gaussian.

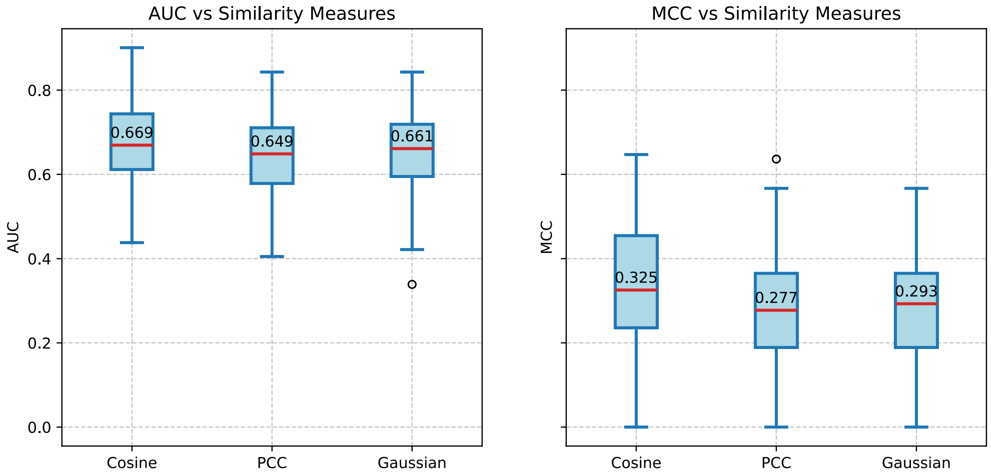

Figure S4: **Similarity measures vs. performance.** AUC (left) and MCC (right) when cross-omics edges are built with cosine, Pearson, or a Gaussian-kernel similarity. Boxplots summarize results across 100 data splits; horizontal red bars mark medians (annotated), boxes indicate interquartile ranges.

S4 Feature Selection: Strategies and Threshold Tuning

We evaluated three strategies for selecting input features: (i) *high-variance filtering*, (ii) *variance-informed correlation* (selecting high-variance features most correlated with labels), and (iii) *minimum redundancy maximum relevance (mRMR)*. As illustrated in Figure S5, across multiple feature budgets, high-variance filtering delivered the strongest and most stable performance (highest median AUC/MCC), variance-informed correlation performed worst, and mRMR fell in between. Intuitively, high-variance features provide higher signal-to-noise in small, high-dimensional omics settings; direct correlation is fragile due to noisy/weak label–feature correlations; and mRMR reduces redundancy but can over-curate by removing co-expressed pathway signals.

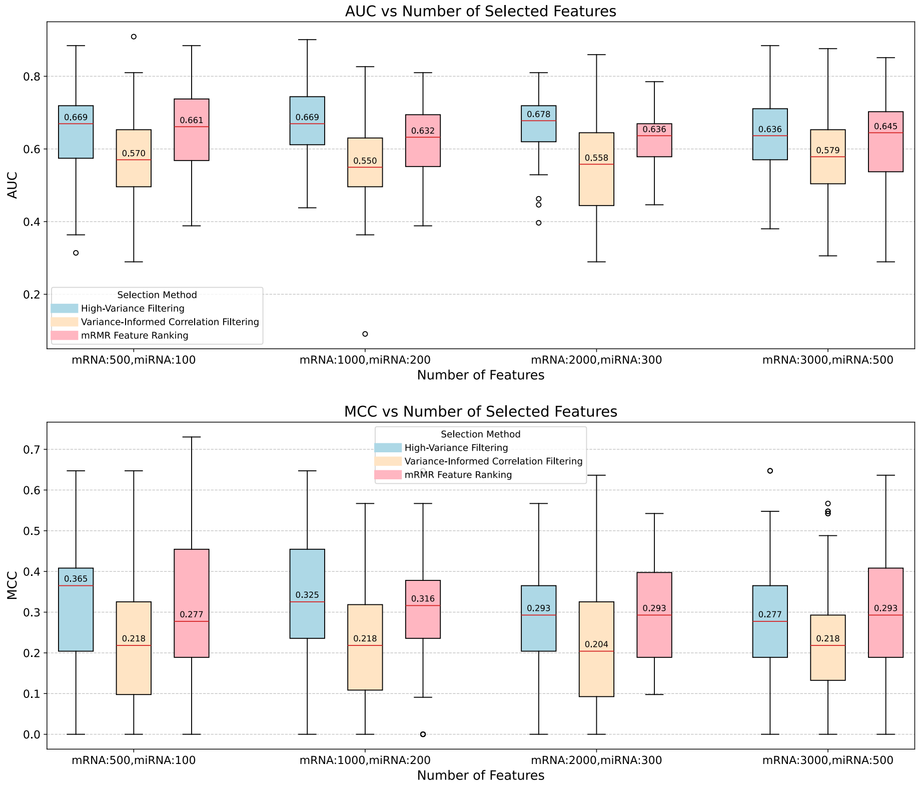

Figure S5: **Different feature-selection strategies vs. performance.** AUC (top) and MCC (bottom) across several feature budgets comparing high-variance filtering, variance-informed correlation, and mRMR. Boxplots summarize results across 100 data splits; horizontal red bars mark medians (annotated), boxes indicate interquartile ranges.

To quantify how the variance cutoff affects performance, we varied the mRNA and miRNA variance thresholds from 0 to 5 in steps of 0.1 on LUAD (Figure S6, top), retaining only features above each cutoff. The best region appears at *lower–to–moderate mRNA thresholds* (typically ≤ 3), which corresponds to keeping at least $\sim$ 1,000 mRNA features. Concretely, for mRNA a threshold of 0.0 retains ∼10,000 features; 1.0: ∼4,000; 2.0: ∼2,000; 3.0: ∼1,000; 4.0: ∼600; 5.0: ∼400. To assess efficiency (Figure S6, bottom), we scaled feature counts around a ∼1k-per-omic baseline to $0.5\times, 1\times, 2\times, 5\times$, and $10\times$: runtime is nearly unchanged up to $\sim 2\times$ and rises sharply beyond. Overall, selecting $\sim$ 1,000–2,000 features per omic provides a good balance between performance and computational cost.

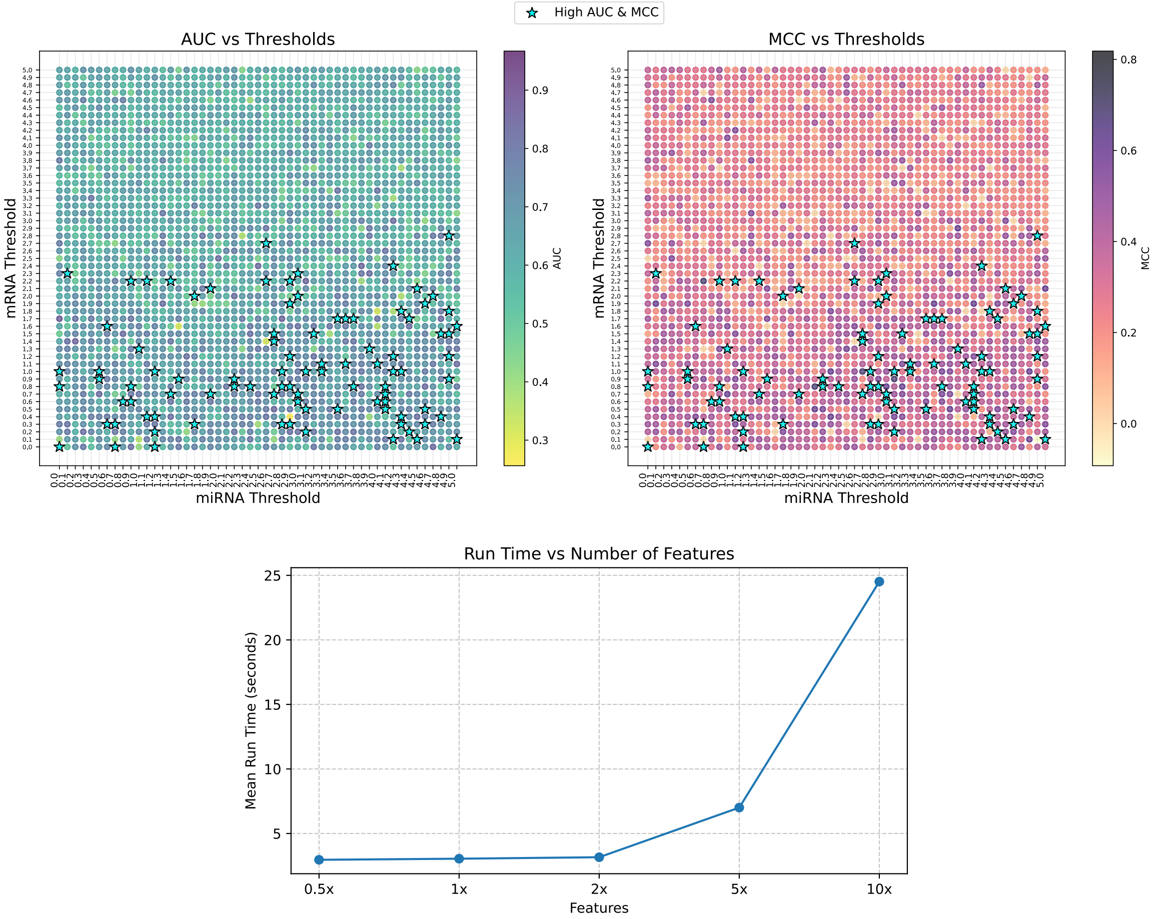

Figure S6: **Performance–efficiency trade-off under feature scaling.** *Top:* AUC (left) and MCC (right) on LUAD as variance thresholds for mRNA (x-axis) and miRNA (y-axis) vary; colors indicate metric values and black stars mark the top 5% settings. *Bottom:* Mean runtime as the number of features is scaled relative to a $\approx 1,000$-per-omic baseline (0.5×, 1×, 2×, 5×, 10×).

S5 Comparing Three Omics Performance Against Baselines

The performance results presented in Table S1 highlight that SynOmics outperforms the other models when trained with the additional DNA methylation data.

		**SynOmics**	**MOGONET**	**MoGCN**	**SUPREME**	**MOGLAM**	**OmicsFormer**	**MOGAT**
Survival	AUC	**0.6810**	0.5958	0.6176	0.6081	0.6007	0.6294	0.6629
Duration	MCC	**0.3360**	0.2605	0.1721	0.1180	0.2688	0.2002	0.2338
Disease-Free	AUC	**0.6478**	0.6041	0.5862	0.4515	0.6314	0.5526	0.5285
Duration	MCC	**0.3391**	0.3039	0.094	0.0009	0.3260	0.0497	0.0152

Table S1: **Classification performance on the LUAD dataset using three omics types.** Metrics: AUC and MCC. Best results per metric are in bold.

S6 Hyperparameter Tuning

In the 'dual alignment' module, the hyperparameter α plays a key role during training. When α is too large, the model primarily focuses on aligning the two hidden representations (intra and inter-omics). On the other hand, if α is too small, the model places more importance on ensuring the hidden representation is close to the original data. In our experiments, as shown in Figure S7, the scores with respect to α initially appear unpredictable. However, upon closer inspection, we can see that the variations in both AUC and MCC are minimal. This suggests that the model maintains robust performance across different values of α.

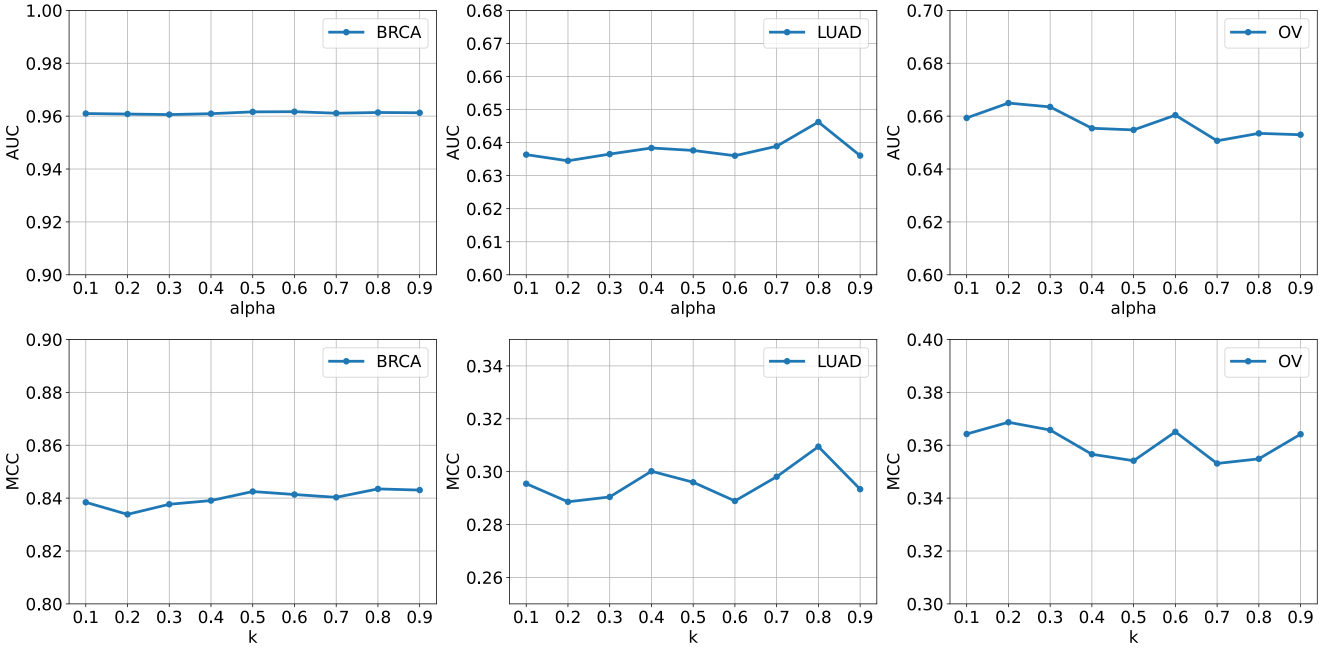

Figure S7: AUC and MCC scores for the BRCA (ER status), LUAD (survival duration), and OV (survival duration) datasets at different values of the 'dual alignment' hyperparameter α, which controls the balance between alignment and reconstruction loss..

We also describe additional hyperparameter settings used for training SynOmics. To identify the optimal configuration, we constructed a parameter grid with multiple candidate values for each hyperparameter and trained the model across 100 random splits. The common hyperparameters shared between both modules of SynOmics, as well as those specific to the dual alignment module, are summarized in Table S2. The hyperparameter combinations for the best 10 models of the 'weighted message passing' module are shown in Table S3. The hyperparameter combinations for the 10 best-performing models of the 'dual alignment' module are shown in Table S4.

	Hyperparameter	Description
Common Parameters	Number of layers	Number of SynOmics layers used during training.
	Batch size	Batch size used for feeding input data during training.
	Learning rate	Learning rate used by the optimizer.
	Epochs	Total number of training epochs.
	Adjacency threshold	Threshold used to binarize the graph adjacency matrix.
	Hidden dimension	Dimensionality of the hidden layer in the fully-connected prediction layer.
	Bias	Indicates whether the model includes a bias term.
WMP Parameters	k	Controls the weight for intra-omics contribution during integration of intra- and inter-omics hidden representations.
DA Parameters	Pretraining epochs	Number of epochs used for pretraining the SynOmics layers before alignment.
	α	Weight assigned to the alignment loss in the combined loss function (alignment + reconstruction).

Table S2: Descriptions of the hyperparameters used to train SynOmics. 'WMP' refers to weighted message passing, and 'DA' refers to dual alignment.

Number of Layers	Batch Size	Learning Rate	Epochs	Adjacency Threshold	Hidden Dimension	Bias	k
1	32	0.001	50	0.1	64	True	0.8
1	32	0.0001	50	0.0	64	True	0.7
1	32	0.001	50	0.5	64	True	0.9
5	32	0.0001	30	0.0	64	True	0.7
3	32	0.001	100	0.0	32	True	0.6
1	32	0.0001	100	0.1	32	True	0.5
2	32	0.0001	100	0.5	64	True	0.9
2	32	0.0001	30	0.5	64	True	0.9
1	32	0.001	30	0.5	64	True	0.8
1	32	0.001	50	0.5	64	True	0.5

Table S3: Top 10 performing model hyperparameters for the 'weighted message passing' module.

Number of Layers	Batch Size	Learning Rate	Epochs	Pretraining Epochs	Adjacency Threshold	Hidden Dimension	Bias	α
3	32	0.001	50	10	0.0	64	True	1.0
1	32	0.001	10	10	0.1	32	True	1.0
1	32	0.001	50	30	0.0	64	True	1.0
1	32	0.001	50	50	0.1	32	True	0.0
1	32	0.001	30	10	0.1	64	True	0.5
1	32	0.001	50	30	0.0	32	True	0.0
1	32	0.0001	50	10	0.1	32	True	0.0
1	32	0.001	30	10	0.0	32	True	0.8
1	32	0.0001	50	10	0.0	32	True	0.8
1	32	0.001	10	30	0.0	64	True	1.0

Table S4: Top 10 performing model hyperparameters for the 'dual alignment' module.

S7 Supplementary Data

We provide three CSV files in the supplementary data. The organization of these files is described below:

1. `Data_S1_latent_stage.csv`. One row per latent feature summarizing its association with clinical stage.
   - `latent`: latent ID
   - `rho_stage`: Spearman correlation between latent score and stage

- `pval`: nominal p-value for `rho_stage`
- `fdr`: FDR (BH) across the 33 preselected latents
- `trend`: orientation vs. stage; `up-with-stage` or `down-with-stage`

2. `Data_S2_latent_gene_correlations.csv`. Complete gene-level associations for every latent (one row per latent–gene pair).

   - `latent`: latent ID
   - `gene`: HGNC symbol
   - `rho`: Spearman correlation between latent score and gene expression
   - `fdr`: FDR (BH) across genes for that latent
   - `gene_stage_trend`: gene's direction vs. stage, combining the latent's stage trend with the gene–latent sign ($\rho > 0 \Rightarrow$ same as latent; $\rho < 0 \Rightarrow$ opposite)

3. `Data_S3_latent_gsea_results.csv`. Full pre-ranked GSEA results per latent–pathway (Reactome 2022), including statistics and leading-edge genes.

   - `latent`: latent ID
   - `pathway`: Reactome term (name/ID)
   - `nes`: normalized enrichment score (signed)
   - `pval`: nominal enrichment p-value
   - `lead_genes`: semicolon-separated leading-edge genes
   - `pathway_direction`: optional convenience label; `nes>0 (up-with-latent)` or `nes<0 (down-with-latent)` (can be mapped to up/down-with-stage using S1 `trend`)
